# Matrix crosslinking enhances macrophage adhesion, migration, and inflammatory activation

**DOI:** 10.1063/1.5067301

**Published:** 2019-03-27

**Authors:** Jessica Y. Hsieh, Mark T. Keating, Tim D. Smith, Vijaykumar S. Meli, Elliot L. Botvinick, Wendy F. Liu

**Affiliations:** Department of Biomedical Engineering and The Edwards Lifesciences Center for Advanced Cardiovascular Technology, University of California Irvine, Irvine, California 92697, USA

## Abstract

Macrophages are versatile cells of the innate immune system that can adopt a variety of functional phenotypes depending on signals in their environment. In previous work, we found that culture of macrophages on fibrin, the provisional extracellular matrix protein, inhibits their inflammatory activation when compared to cells cultured on polystyrene surfaces. Here, we sought to investigate the role of matrix stiffness in the regulation of macrophage activity by manipulating the mechanical properties of fibrin. We utilize a photo-initiated crosslinking method to introduce dityrosine crosslinks to a fibrin gel and confirm an increase in gel stiffness through active microrheology. We observe that matrix crosslinking elicits distinct changes in macrophage morphology, integrin expression, migration, and inflammatory activation. Macrophages cultured on a stiffer substrate exhibit greater cell spreading and expression of αM integrin. Furthermore, macrophages cultured on crosslinked fibrin exhibit increased motility. Finally, culture of macrophages on photo-crosslinked fibrin enhances their inflammatory activation compared to unmodified fibrin, suggesting that matrix crosslinking regulates the functional activation of macrophages. These findings provide insight into how the physical properties of the extracellular matrix might control macrophage behavior during inflammation and wound healing.

## SIGNIFICANCE STATEMENT

Macrophages are essential cells of the innate immune system which dynamically respond to signals in their environment to promote inflammation and tissue remodeling. In this study, we examine how physical changes in the extracellular matrix (ECM) alter macrophage adhesion and function. Culture of macrophages on photo-crosslinked ECM hydrogels shows that crosslinking leads to enhanced macrophage spreading, integrin-mediated adhesion, migration, and inflammatory activation. This work has a broad impact on the role of the ECM on macrophage function during healing after injury or tissue remodeling during disease.

## INTRODUCTION

Macrophages are innate immune cells that are central to many biological processes including development, metabolism, and tissue homeostasis.^1^ These cells are also dynamic regulators of the wound healing process, advancing and resolving inflammation in response to cues in their microenvironment.^2^ Macrophages are recognized for their remarkable plasticity and can assume a diverse range of phenotypes depending on cues from their microenvironment.^3^ In response to pathogens or damaged cells, macrophages adopt a classically activated, pro-inflammatory phenotype to promote inflammation. However, in the presence of wound healing cytokines such as interleukin-4 (IL-4) and interleukin-13 (IL-13), macrophages polarize toward a pro-regenerative phenotype critical for tissue repair. Macrophage phenotypes are clearly regulated by soluble cues in their environment, but the role of adhesive and physical cues from the extracellular matrix (ECM) remains less well defined.

Studies from the biomaterial and bioengineering community over the past decade have contributed significantly to our knowledge about how physical cues including material topography and rigidity influence macrophage functions.^4–7^ In addition, ECM-based biomaterials are thought to promote wound healing phenotypes.^8,9^ However, how the physical properties of the ECM contribute to macrophage behavior has not been well-characterized. Wound healing is associated with dynamic changes in the composition and physical properties of the ECM, as the matrix is crosslinked or degraded during tissue remodeling.^10,11^ The initial provisional extracellular matrix, fibrin, is formed by polymerization of soluble plasma fibrinogen with the enzyme thrombin, which is activated during injury.^12^ Fibrin not only is hemostatic but also provides a matrix for the influx of neutrophils and macrophages as they home to the site of injury to clear infectious agents and damaged tissue.^13,14^ Our group previously showed that culture of macrophages on fibrin inhibits their inflammatory activation when compared to culture on standard polystyrene or glass surfaces. Here, we asked whether reduced inflammatory activation of cells on fibrin may be attributed to the physical properties of this material and specifically if manipulating the stiffness of the matrix through crosslinking would alter the activity of macrophages.

Here, we used a photo-crosslinking method to enhance the rigidity of fibrin hydrogels and examined macrophage adhesion, motility, and activation upon culture on crosslinked versus control non-crosslinked matrices. We found that macrophages cultured on photo-crosslinked matrices exhibited increased spreading and expression of αM integrin, their major integrin subtype. In addition, macrophages cultured on crosslinked matrices exhibited enhanced motility and lipopolysaccharide (LPS)-induced tumor necrosis factor alpha (TNF-α) secretion when compared to cells cultured on non-crosslinked fibrin gels. Together, our data suggest that matrix crosslinking alters macrophage adhesion and function and may play a role in regulating macrophage behavior during tissue remodeling and healing.

## RESULTS AND DISCUSSION

### Matrix crosslinking increases fiber density and rigidity

**FIG. 1.**
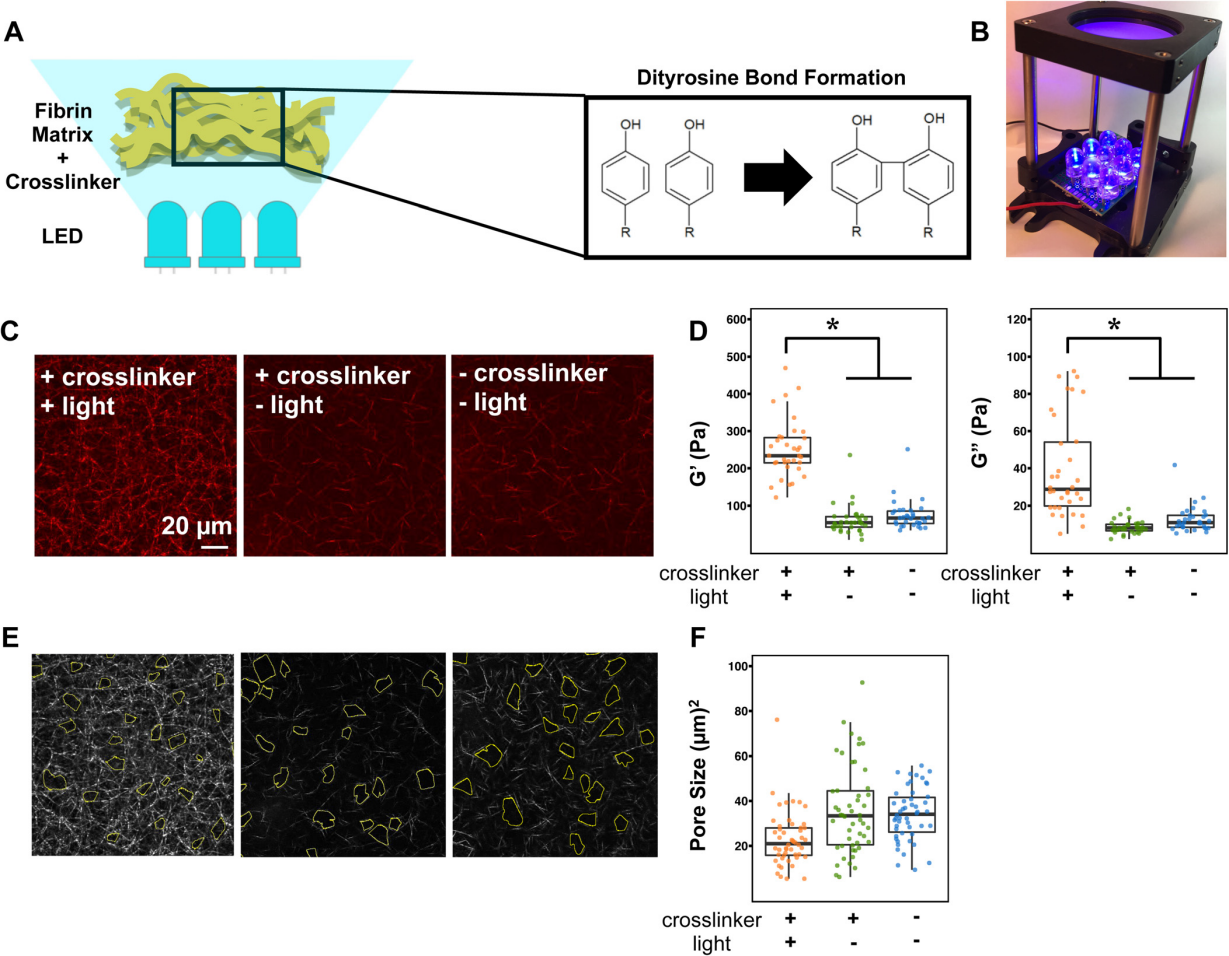
Characterization of ruthenium-based photo-crosslinked fibrin gels. (a) Schematic detailing the photo-crosslinking process. (n) 465 nm blue light is provided by a light rig fabricated in-house. (c) Representative backscatter images of 2 mg/ml fibrin gels with indicated conditions. Scale bar: 20 *μ*m. (d) Scatterplot of AMR measurements of G′ in 2 mg/ml fibrin gels with the indicated conditions. The asterisk denotes p < 0.05 using the Mann Whitney test with a Bonferroni correction. (e) Corresponding representative analysis images of the pore size of 2 mg/ml fibrin gels with indicated conditions. (f) Scatterplot of pore size measurements in 2 mg/ml fibrin gels with the indicated conditions.

We introduced dityrosine bonds to fibrin hydrogels using ruthenium II trisbipyridyl chloride [RuII(bpy_3_)]^2+^ and sodium persulfate (SPS) through a photo-induced method.^15–17^ In the presence of SPS, the ruthenium metal complex is activated by blue light, resulting in a reactive tyrosine radical as well as a sulfate radical—the intermediate radicals react with another tyrosine phenyl group to form a covalent dityrosine bond [Figs. 1(a) and 1(b)]. This methods takes advantage of the fact that tyrosines are abundantly found in fibrinogen—the β-chain, γ-chain, and α-chain have 4.9% tyrosine, 5.6% tyrosine, and 0.65% tyrosine, respectively. Therefore, the technique requires no further modification of fibrinogen and has been utilized to make fibrin a mechanically stronger surgical tissue sealant and for fibrin-based tissue engineering applications, without causing significant cell death.^16,18,19^

To characterize the material properties of the crosslinked matrix, we used laser scanning confocal microscopy to examine fiber architecture and active microrheology (AMR) to assess gel stiffness [Figs. 1(c)–1(f)]. 2 *μ*m diameter probe microbeads were introduced into the unpolymerized fibrinogen solution and embedded into the gels during polymerization. Optical tweezers forced oscillations of the microbeads confined within the gel, and a detection laser determined the beads' positions. Bead oscillations were used to determine the bulk modulus, G′, locally around the individual beads using a previously described method.^20,21^ We determined that a non-crosslinked fibrin gel had an averaged G′ value of 74.2 ± 39.5 Pa, and crosslinking increased the G′ value to 251.7 ± 77.7 Pa [Fig. 1(d)]. Thus, a ruthenium photo-crosslinked gel is significantly stiffer than a non-crosslinked fibrin gel. Analysis of the fiber network structures in non-crosslinked fibrin gels vs. the photo-crosslinked fibrin demonstrates a denser structure with smaller pore sizes [Figs. 1(c), 1(e), and 1(f)]. However, the fiber diameter and length appear to be similar between the two conditions. The crosslinking solution by itself, without exposure to blue light, has no effect on altering the network architecture of the fibrin gel. Together, these characterization studies demonstrated that photo-crosslinking of fibrin gels using ruthenium and sodium persulfate in the presence of blue light generated a mechanically stiffer fibrin gel with decreased pore sizes.

### Matrix crosslinking enhances cell spreading and αM integrin expression

**FIG. 2.**
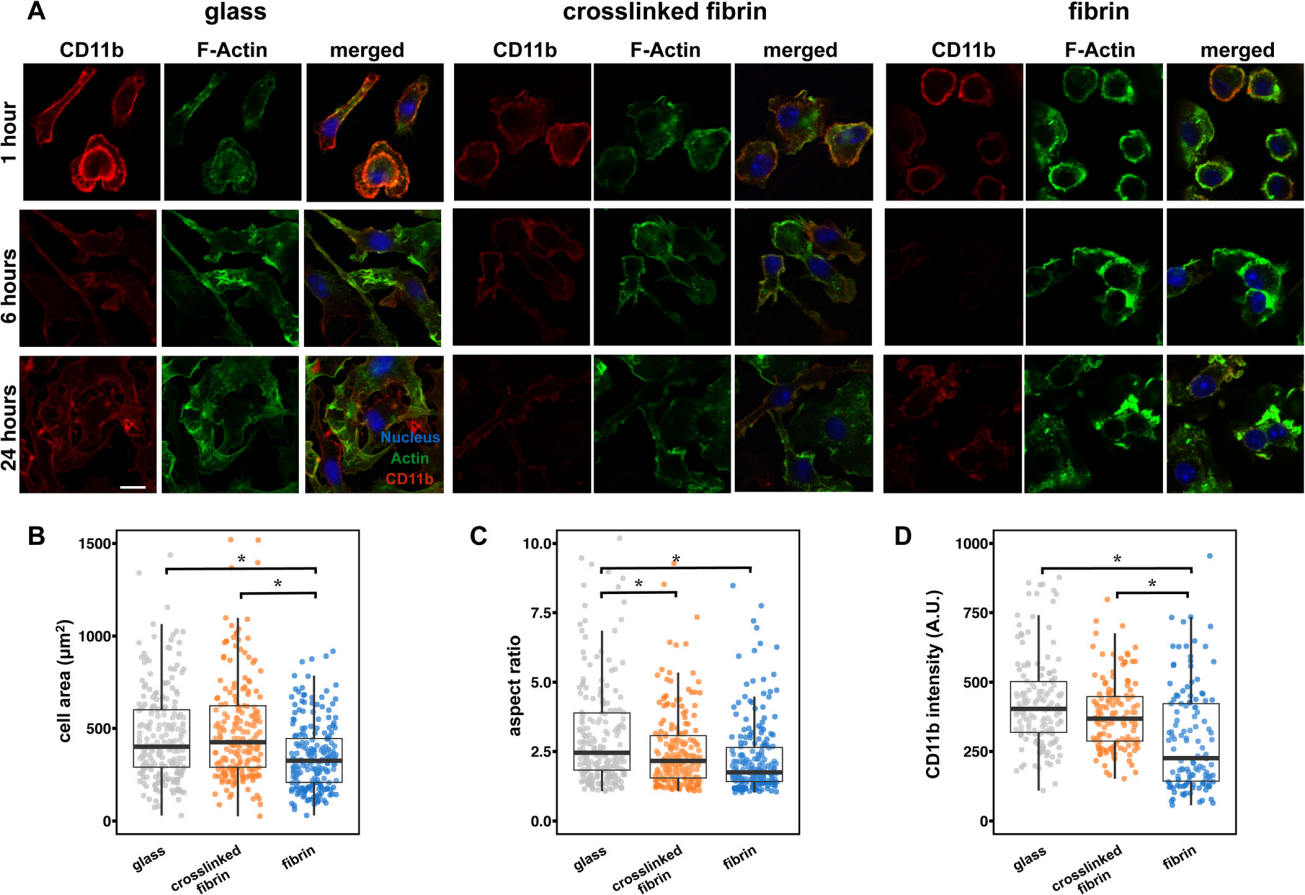
Macrophage spread area and integrin expression are enhanced on crosslinked fibrin. (a) Fluorescence images of αM (left), F-actin filaments (center), and merged images (right) of BMDMs cultured on glass, crosslinked fibrin gels, or non-crosslinked fibrin gels at 1, 6, and 24 h after seeding. The scale bar is 10 *μ*m. (b) Scatterplot of the cell area (*μ*m) of cell culture on indicated conditions fixed after 6 h of adhesion. (c) Scatterplot of the elongation factor (length of the major axis divided by the length of the minor axis) of cell culture on indicated conditions fixed after 6 h of adhesion. (d) Scatter plot of αM intensity of cell culture on indicated conditions fixed after 6 h of adhesion. Over 50 [for (b) and (c)] or 40 (d) cells were analyzed per condition across 3 biological replicates. The asterisk denotes p < 0.05 by one-way ANOVA followed by Tukey's HSD post-hoc test.

To determine the effects of crosslinking on macrophage adhesive interactions with the matrix, we examined morphology and integrin expression of murine bone marrow derived macrophages (BMDMs) cultured on crosslinked and non-crosslinked gels and also compared with those of the cells cultured on glass. We first evaluated morphology by staining the actin cytoskeleton with phalloidin over a time course by immunofluorescence microscopy [Fig. 2(a)]. We observed more cortical actin features in cells cultured on fibrin and dynamic spreading and adhesion over time in all conditions. We further quantified the cell area and aspect ratio at 6 h after seeding, the time point at which migration was evaluated and macrophages were stimulated in later studies. At this time point, macrophages cultured on non-crosslinked fibrin remain round and clustered, with a spread cell area of 350 *μ*m^2^ [Fig. 2(b)]. Macrophages seeded on glass are more well spread, averaging approximately 500 *μ*m^2^, and cells cultured on crosslinked fibrin gels were also highly spread, exhibiting some filopodial extensions. Quantification of the aspect ratio, or the longest (major) axis divided by the short (minor) axis, showed that macrophages had the highest aspect ratio on glass, averaging around 3.4. Compared to cells cultured on glass, cells cultured on non-crosslinked fibrin gels had a significantly lower average aspect ratio of 2.2 and macrophages on crosslinked fibrin gels exhibited an intermediate aspect ratio of 2.7 [Fig. 2(c)]. Together, these data suggest that macrophages are more spread and less round on crosslinked fibrin gels when compared to cells cultured on non-crosslinked fibrin gels.

We next investigated whether the expression level of integrins, cell surface receptors that bind to ECM proteins, was modified by matrix crosslinking. We investigated the expression of αM integrin, or CD11b, because it is the most highly expressed macrophage integrin and is thought to be the primary integrin that mediates adhesion to fibrin.^22,23^ We analyzed αM integrin on the surface on macrophages by fixing cells without permeabilization and immunostained over a time course after adhesion on different surfaces [Fig. 2(a)]. We found that the expression of αM integrin was higher on glass, particularly at the earlier time points, and that cells cultured on non-crosslinked fibrin gels expressed less αM integrin. This difference was confirmed by flow cytometry, where we observed that culture on fibrin, as well as collagen, led to almost a two-fold reduction in αM expression, among cell surface receptors (supplementary material, Fig. 1). In contrast, BMDMs on crosslinked fibrin expressed higher levels of αM integrin at 6 h after adhesion although the expression levels decrease over time. Quantification of αM integrin intensity per cell at 6 h after adhesion showed that average αM integrin intensity on glass is similar to that observed on crosslinked fibrin [Fig. 2(d)]. Overall, the level of αM integrin was highly heterogenous with a broad range of individual cell expression within each condition and dynamic as the cells adhered over the course of the time frame examined. Most of the αM integrin expression was localized on the periphery of the cells across all conditions. These results suggest that despite the presence of αM integrin binding sites within fibrin, the expression levels are low. Furthermore, robust expression of αM integrin may be potentiated by features associated with culture on a glass surface, such as adsorbed ECM proteins or substrate rigidity.

### Matrix crosslinking increases motility of macrophages

**FIG. 3.**
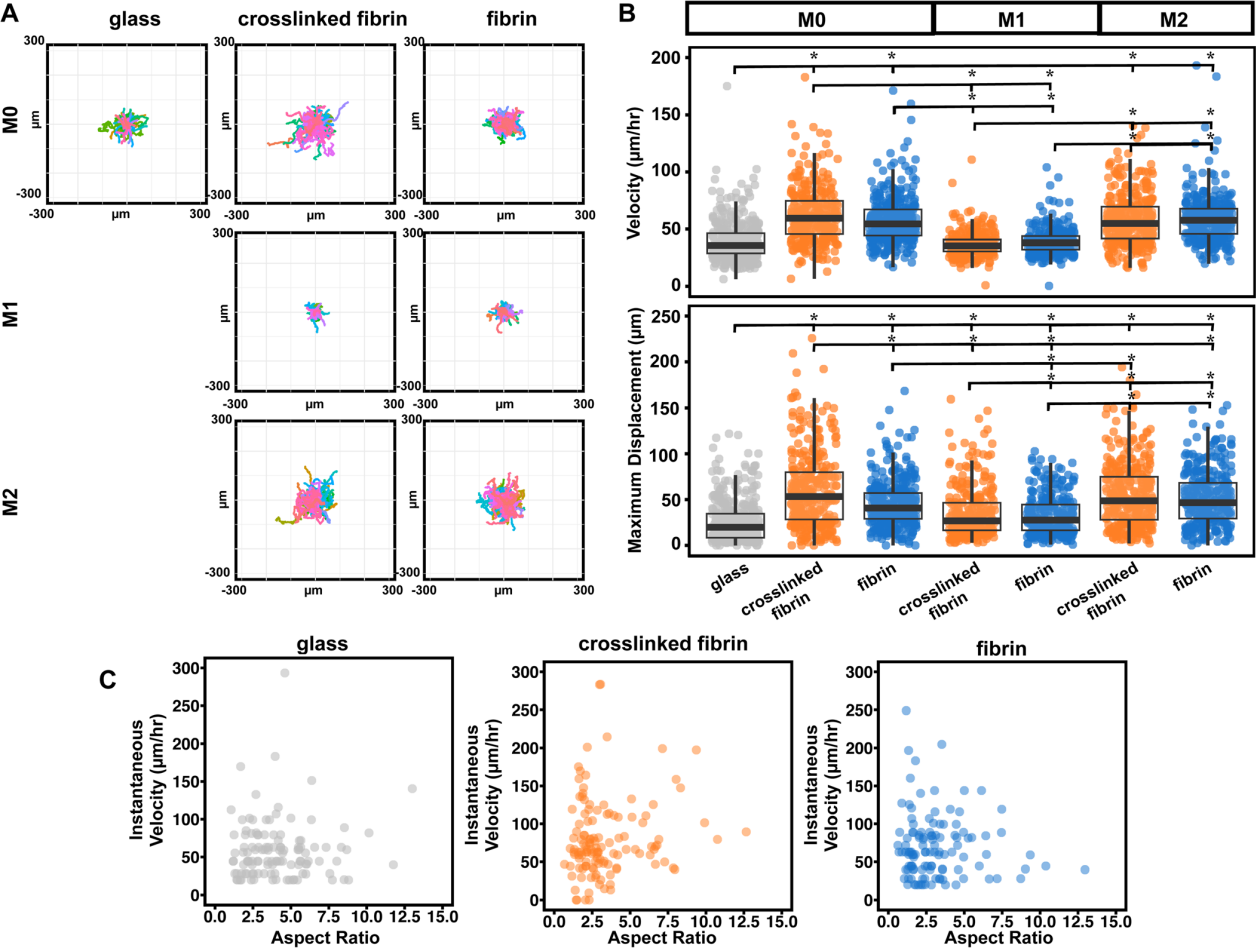
Macrophage motility increases upon matrix crosslinking. (a) Representative displacement plots of unstimulated, LPS and IFN-γ, and IL-4 and IL-13 stimulated macrophages on glass, crosslinked, or non-crosslinked surfaces. (b) Scatter plots of velocity (left) or maximum displacement (right). Bar and whisker plot values are mean ± SEM of at least 300 cells across 4 biological replicates for each condition. The asterisk denotes p < 0.05 by one-way ANOVA followed by Tukey's HSD post-hoc test. (c) Scatter plots of instantaneous velocity against the aspect ratio of macrophages migrating on glass, crosslinked, or non-crosslinked surfaces. Each dot represents a single cell; at least 90 cells across 2 biological replicates were analyzed for each condition.

To begin to evaluate how matrix crosslinking influences the function of macrophages, we first examined their migration behavior. Macrophages are highly motile cells and need to migrate within tissues for immune surveillance and response to pathogens or damage.^24^ Macrophages are thought to exhibit intermediate migration speeds, faster than fibroblasts and epithelial cells but slower than neutrophils and other leukocytes.^25,26^ Integrins, particularly αMβ2 or CD11b/CD18, have been shown to regulate cell motility in many immune cells, both within tissue and through the endothelium.^27–29^ In macrophages, genetic inactivation of αMβ2 inhibited macrophage efflux from the peritoneal mesothelium to the lymphatics.^30^ We found that BMDMs displaced farther from their starting positions and exhibited higher velocities when cultured on fibrin gels, when compared to cells on glass (Fig. 3 and supplementary material, Figs. 2–4), suggesting that perhaps motility is enhanced on a ECM matrix compared to a very stiff 2D surface. BMDMs migrated at an average velocity of 40 *μ*m/h on glass, and BMDMs on non-crosslinked and crosslinked fibrin gels exhibited average velocity values of 58 and 62 *μ*m/h, respectively [Fig. 3(b)]. In addition, we observed both ameboid and mesenchymal migration modes on all substrate conditions (supplementary material, Figs. 2–4), and also found that cells with higher aspect ratios tended to migrate more slowly and vice versa [Fig. 3(c)]. Interestingly, BMDMs were the most motile on crosslinked fibrin surfaces, in terms of both velocity and maximum displacement. Macrophages on crosslinked fibrin gels displaced up to an average maximal distance of 61 *μ*m, while macrophages on non-crosslinked fibrin gels displaced 45 *μ*m from its origin and only 26 *μ*m on glass. A potential explanation for increased motility on crosslinked fibrin gels is that this matrix provides a denser fibrillar architecture and greater mechanical stiffness, both of which may be needed for enhanced migration. Stimulation of macrophages with LPS and interferon gamma (IFN-γ) to induce “M1” activation reduced the velocity and maximum displacement on both crosslinked and non-crosslinked fibrin matrices, whereas stimulation with IL-4 and IL-13 to induce “M2” polarization had no effect on velocity and moderately increased displacement on non-crosslinked fibrin matrices [Figs. 3(a) and 3(b), supplementary material, Fig. 5]. These data suggest that activation with soluble signals has a greater effect on macrophage migration compared to their matrix environment.

**FIG. 4.**
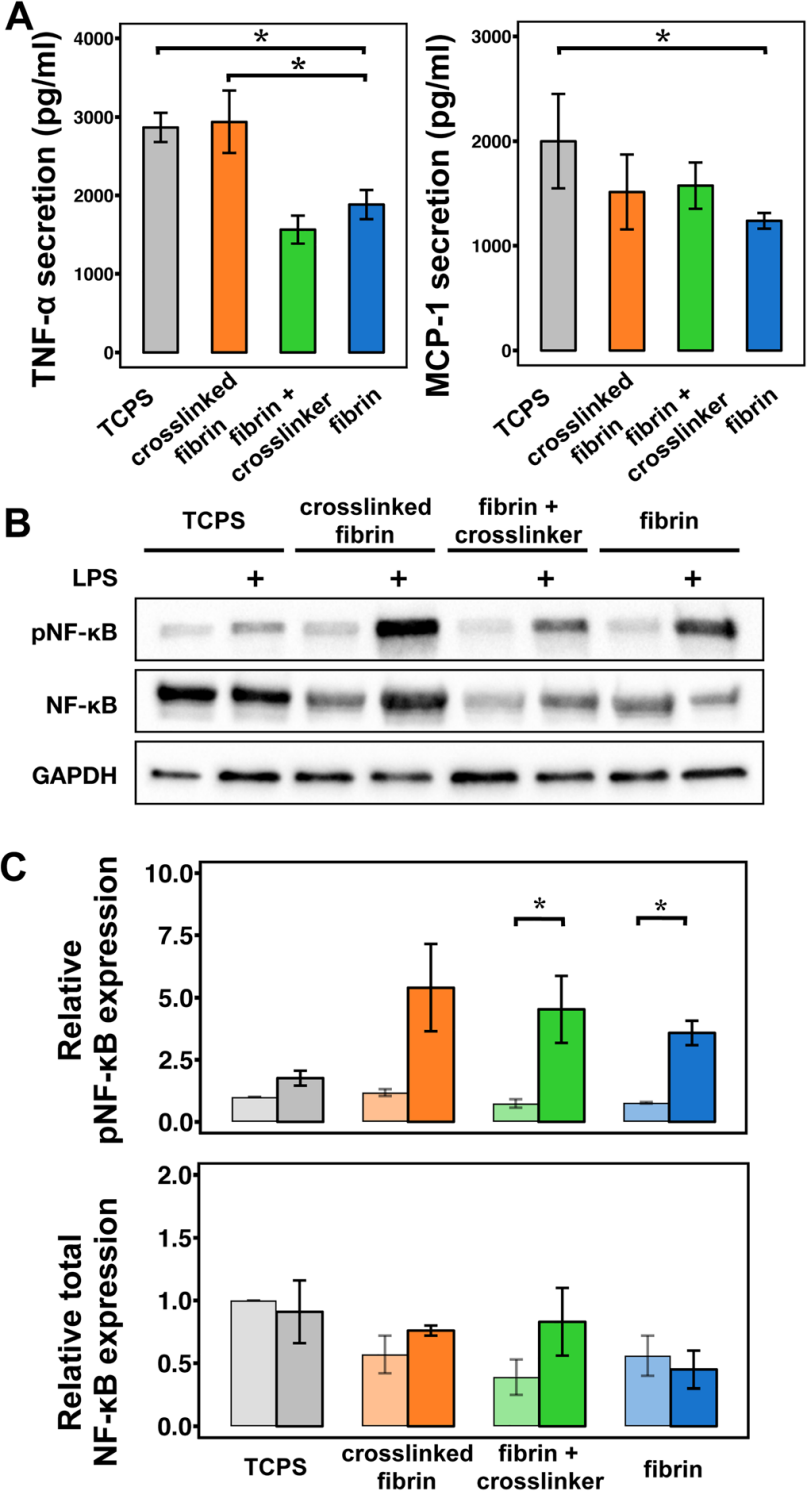
Macrophage inflammatory activation increases upon matrix crosslinking. (a) Graph of TNF-α and MCP-1 secretion by macrophages cultured on indicated fibrin gel conditions relative to the tissue culture polystyrene surface (TCPS). BMDMs were stimulated at 6 h after seeding with 5.0 ng/ml of UltraPure LPS and 1.0 ng/ml of IFN-γ. The values are mean ± SEM of n = 6 biological replicates for TNF-α and n = 5 biological replicates for MCP-1. Asterisks denote p < 0.05 by Student's t-test followed by false discovery rate correction for multiple comparisons. (b) Representative Western blot of phospho-NF-κB p65, NF-κB, and GAPDH of unstimulated or LPS and IFN-γ macrophages cultured on the indicated surface or fibrin conditions. (c) Quantification of average phospho-NF-κB p65 (top) and total NF-κB (bottom) across two separate experiments. Lighter shaded bars are unstimulated macrophages, while darker shaded bars are LPS and IFN-γ stimulated macrophages. Asterisks denote p < 0.05 by unpaired Student's t-test. Error bars indicate the SEM.

**FIG. 5.**
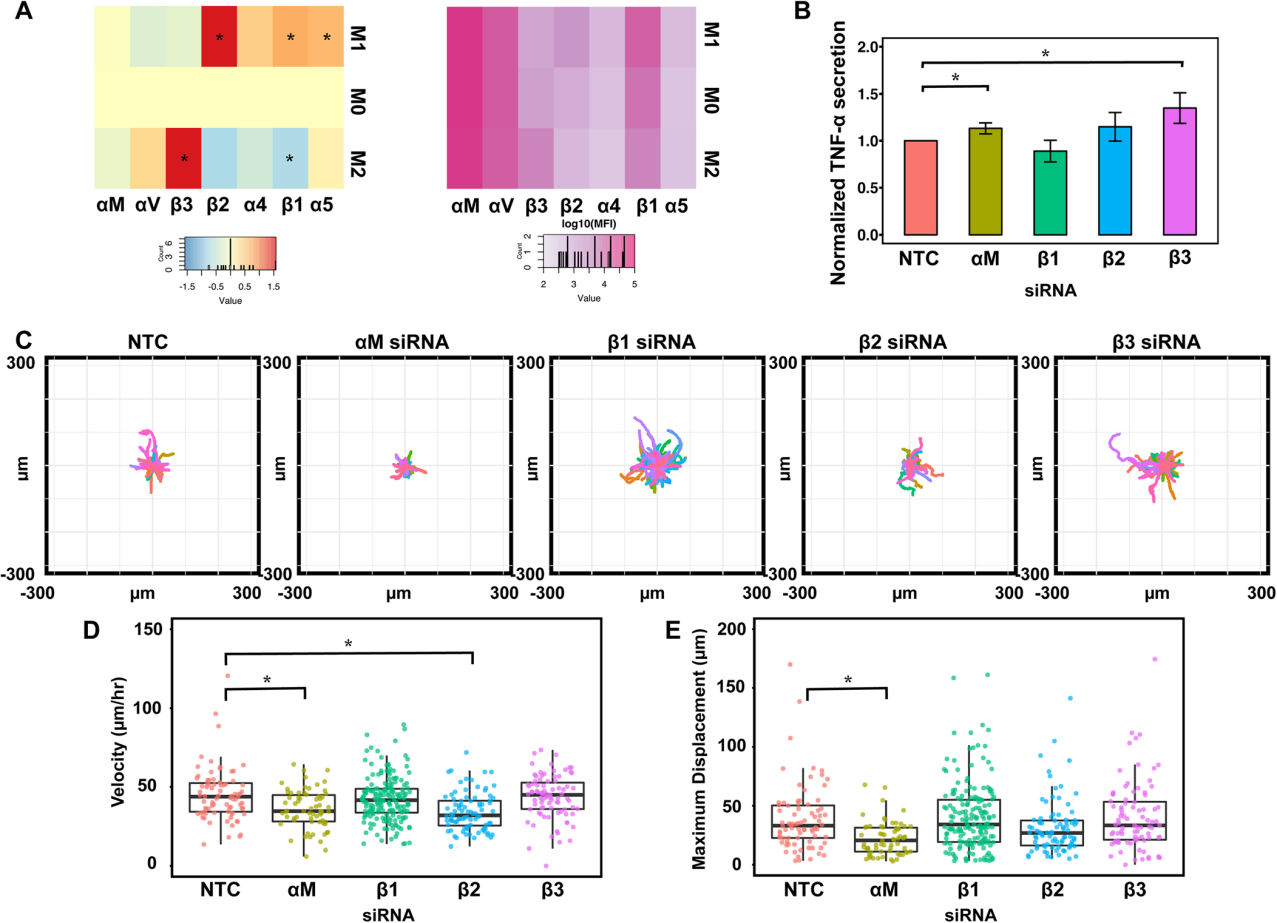
Role of integrins in macrophage inflammatory activation and migratory behavior. (a) Median fluorescence intensity of flow cytometry data, expressed as log2 fold expression vs untreated macrophages. Asterisks indicate significant differences vs unstimulated by the pairwise t-test, FDR < 0.05. Plot to illustrate approximate relative abundance by plotting the log10 absolute value of median fluorescence intensity of flow cytometry data for integrins. (b) Graph of relative TNF-α secretion by BMDMs after knockdown of indicated integrin genes using siRNAs. (c) Representative displacement plots of macrophages on glass with indicated integrin knocked down. (d) Scatter plots of velocity (e) or maximum displacement. Bar and whisker plot values are mean ± SEM of at least 90 cells across 2 biological replicates for each condition. The asterisk denotes p < 0.05 by one-way ANOVA followed by Tukey's HSD post-hoc test.

Our data show that an increased matrix crosslinking enhances the motility of unstimulated macrophages. Given that crosslinked matrices have higher shear modulus [Fig. 1(d)], these data are consistent with a study showing that human-monocyte derived macrophages migrated faster on stiff (280 kPa) when compared to soft (3 kPa) substrates.^31^ It is possible that the differences are due to changes in matrix density, as has been previously reported to influence macrophage migration.^32,33^ Interestingly, our results differ with an earlier study showing that Factor XIII-induced crosslinking reduced motility within fibrin.^13,14^ This previous work studied cells migrating through the gel, and it is possible that migration through as opposed to on top of the gel, or the crosslinking method used, contributed to the differences observed.

### Matrix crosslinking enhances macrophage inflammatory activation

Substrate rigidity has been associated with increased inflammatory cytokine secretion by murine macrophages.^34,35^ We also showed that culture of BMDMs on soft fibrin gels abrogated TNF-α secretion in response to LPS and IFN-γ when compared to cells cultured on a stiff polystyrene surface.^36^ To more specifically examine the effect of fibrin matrix crosslinking on murine BMDM inflammatory activation, we cultured macrophages on fibrin gels that were crosslinked, exposed to only the crosslinker solution but not exposed to light, or a non-crosslinked gel, and also compared with tissue culture polystyrene (TCPS). At 6 h after seeding, we stimulated cells with LPS and IFN-γ for 12 h and then collected the supernatants to investigate cytokine secretions. Confirming what we had observed in our earlier work, we observed an inhibition of inflammatory activation when cells were cultured on fibrin gels when compared to polystyrene [Fig. 5(a)]. This general effect was also observed in cells cultured on collagen matrices, suggesting that inhibition of inflammation may not be specific to fibrin (supplementary material, Fig. 6). We found that macrophages cultured on crosslinked fibrin exhibited higher secretion of TNF-α when compared to cells cultured on non-crosslinked fibrin. Somewhat surprisingly, the level of TNF-α secreted by cells cultured on crosslinked fibrin was similar to that of cells cultured on polystyrene, even though these two materials differ dramatically in elasticity (∼GPa for polystyrene). The secretion level from cells cultured on fibrin that was exposed to crosslinker without light was similar to fibrin alone. Evaluation of macrophage chemoattractant protein 1 (MCP-1, also known as CCL2), an inflammatory chemokine, showed decreased levels in cells cultured on fibrin, but not crosslinked or crosslinker containing fibrin gels, compared to those on polystyrene [Fig. 4(a)].

While TNF-α and MCP-1 represent two inflammatory cytokines, we sought to investigate whether the matrix environment regulates inflammation more broadly. To address this, we probed nuclear factor kappa B (NF-κB), a transcription factor that is phosphorylated and activated upon stimulation with inflammatory signals including LPS. We found that culture of cells on fibrin inhibited their expression of NFκB when compared to cells on polystyrene and that crosslinking enhanced the expression relative to non-crosslinked gels [Figs. 4(b) and 4(c)]. Phosphorylation of NF-κB upon stimulation with LPS and IFN-γ followed a similar trend. However, introduction of the crosslinker itself also induced a moderate increase in NF-κB levels and its phosphorylation, suggesting that the ruthenium itself may influence this inflammatory transcription factor. Nonetheless, these data together suggest that macrophage inflammatory activation on ECM is dependent on matrix crosslinking, which elicit changes in the stiffness, pore size, and fibril architecture.

### Role of integrins in macrophage activation and migration

Our studies showed that macrophages cultured on crosslinked matrices expressed higher levels of αM integrin [Figs. 2(a) and 2(c)], motility (Fig. 3), and inflammatory activation (Fig. 4). To investigate the role of integrins in motility and inflammatory activation, we first evaluated the expression of a panel of integrins including αM, αV, α4, α5, β1, β2, and β3 integrins and found that αM was the most highly expressed integrin followed by αV and β1 subtypes and that several integrins are differentially regulated by M1 and M2 stimulation [Fig. 5(a)]. Knockdown of a subset of these integrins led to enhanced inflammatory activation, suggesting that integrins inhibit inflammatory activation [Fig. 5(b) and supplementary material, Fig. 7]. These data corroborate with some reports but are distinct from others.^37–39^ These data suggest that the increased inflammatory activation of macrophages on crosslinked matrices may not be potentiated by the higher levels of αM integrins observed. It is possible that other integrin subtypes, other molecules such as mechanosensitive ion channels or cytoskeletal regulation, may be involved. In contrast, knockdown of αM integrin inhibited macrophage motility, suggesting that the higher levels observed on crosslinked gels could play a role in enhanced motility [Figs. 5(c) and 5(d)]. Given the presence of αM integrin binding sites found within fibrin, it is possible that this integrin subtype is involved in promoting migration on fibrin. Furthermore, it is possible that crosslinking-induced increases the rigidity and ligand density, facilitating migration through integrin binding interactions.

## CONCLUSIONS

In this study, we examined the effects of matrix crosslinking on macrophage morphology, motility, and activation using a natural ECM substrate. We used a ruthenium photo-crosslinking method to form dityrosine bonds in fibrin gels and enhanced their fiber density and mechanical stiffness. Our findings corroborate with other studies using synthetic matrices including polyethylene glycol and polyacrylamide, where increasing stiffness also elicited greater spreading,^34,40,41^ protrusive actin structures,^41,42^ and increased inflammatory activation.^34,35^ We further characterized integrin expression and migratory behavior and show that increased fibrin crosslinking led to increased αM integrin expression and motility although glass substrates caused an increase in αM integrin and a decrease in motility. While our findings suggest that αM integrin expression is associated with higher levels of inflammatory activation, further investigation of the role of αM integrin suggests that it plays an inhibitory role in inflammatory activation and thus may not be responsible for changes in inflammation caused by matrix crosslinking. Evidence in the literature suggests heightened αM integrin expression is associated with inflammation,^43^ yet in other studies, reduced αM integrin has been shown to lead to increased inflammation.^37,44^ It is possible that the complex and dynamic role of integrins in both cell motility and inflammatory activation may be at play. Furthermore, it is also possible that expression of proteases that influence matrix properties could influence macrophage responses. Nonetheless, our study provides insight into the role of matrix crosslinking and stiffness in macrophage behavior during inflammation and wound healing.

## METHODS

### Ruthenium-based photo-crosslinking

Fibrin gels were fabricated at 2.0 mg/ml using bovine fibrinogen (Calbiochem, EMD Millipore) mixed with 0.4 U of bovine plasma thrombin (Sigma) per mg of protein. Gels were incubated in a humidified, 37 °C environment for 30 min prior to the addition of the crosslinker. The crosslinker solution was composed of 1.5 mg/ml ruthenium II trisbipyridyl chloride (Sigma) and 2.4 mg/ml sodium persulfate (SPS) solution (Sigma) resuspended in Millipore water.^18^ Gels were incubated for 10 min with the crosslinker prior to the exposure to visible light at wavelengths 465 ± 5 nm using a custom built light emitting diode (LED) light apparatus for 20 s. Immediately after exposure, the crosslinking solution was rinsed four times with phosphate buffered saline (PBS) at 37 °C on a shaker and then left in PBS overnight for a fifth wash. Controls that were incubated with just the crosslinker solution were also rinsed in the same way.

### Active microrheology (AMR)

2 mg/ml fibrin gels were polymerized as described previously but with the addition of 2 *μ*m microbeads. 8 *μ*l of beads (0.08% w/v, Bangs Laboratories) were added to unpolymerized fibrinogen to make a 1 ml solution prior to mixing with thrombin. We utilized reflection confocal microscopy to confirm that beads were confined within the fibrin gels. Approximately 30 microbeads were probed via active microrheology per sample. The trapping microbeam that oscillated microbeads, steered by a pair of galvanometer mirrors (Thorlabs), was generated by a continuous-wave fiber laser with emission at 1064 nm (IPG Photonics). A second low power stationary laser at 785 nm (World Star Technologies) is deflected by the probe particle allowing for the measurement of the particle's position. Sinusoidal oscillations of the trapping beam at an amplitude of 100 nm at a frequency of 50 Hz were utilized. G′ and G″ were computed from the amplitude-phase response of each microbead relative to the laser. Pore boundaries were manually traced, and area values were obtained using ImageJ software. Approximately 50 pore areas were traced per sample. Statistical analysis was performed using a Mann-Whitney Test with p < 0.05 with a Bonferroni correction.

### Laser scanning confocal microscopy

For imaging, gels were fabricated on 35 mm glass bottom dishes. Laser scanning confocal back reflection microscopy (backscatter) was performed using an Olympus IX81 microscope. Samples were illuminated with a 559 nm laser light (NTT Electronics Optiλ) using a 40× objective (Olympus) The backscattered light signal was detected using a photomultiplier tube and captured using Olympus Fluoview software.

### Cell culture

Femurs from 6 to 12 weeks old female C57BL/6J mice (Jackson Laboratory) were harvested. Bone marrow from each bone was flushed with Dulbecco's Modified Eagle's medium (DMEM) supplemented with 10% heat-inactivated fetal bovine serum (FBS). The cell pellet was treated with ammonium-chloride-potassium (ACK) lysis buffer (Thermo Fisher) to lyse red blood cells, centrifuged, resuspended, and cultured in D-10 media. D-10 media consist of high-glucose DMEM supplemented with 10% heat-inactivated FBS, 2 mM L-glutamine, 100 U/ml penicillin, 100 *μ*g/ml streptomycin (Thermo Fisher), and 10% conditioned media from CMG 14–12 cell expressing recombinant mouse macrophage colony stimulating factor (M-CSF) produced in-house to induce differentiation to bone marrow derived macrophages (BMDMs).

### Immunofluorescence staining

Macrophages were incubated on materials for 1, 6, and 24 h, then fixed with 4% paraformaldehyde (Electron Microscopy Sciences), and washed with PBS. Samples were blocked with 2% bovine serum albumin in PBS. Samples were incubated with rat anti-αM (M1/70 clone, BioLegend) primary antibody, followed by Alexa Fluor-596 donkey anti-rat secondary antibody (Jackson ImmunoResearch). For actin images, samples were incubated with Alexa Fluor-488 conjugated phalloidin and counterstained with Hoechst 33342 (both from ThermoFisher). Images were acquired using a Zeiss LSM780 confocal microscope using a 63× oil immersion lens and Zen microscope control software.

### Morphology analysis

To assess cell morphology, phalloidin images were utilized. Cell boundaries were manually traced using ImageJ software. The values of the area, major axis, and minor axis were measured and obtained from the software. The aspect ratio was calculated as the major axis, or the longest length of each cell, divided by the minor axis, defined as the length across the nucleus, in a direction that is perpendicular to the long axis. A total of at least 150 cells were analyzed per condition across three separate biological experiments. Statistical analysis was performed using one-way analysis of variance (ANOVA) followed by Tukey's Honestly Significant Difference (HSD) post-hoc test.

### Live imaging of macrophage migration

8-well chamber slides (Thermo Scientific Lab-Tek) were used for live imaging. BMDMs were seeded at 20 000 cells per well in D10 media for 6 h and then placed on a stage incubator. Chambers were observed using an Olympus IX-83 inverted microscope equipped with a Tokai Hit stage incubator and controlled by Micro-Manager. Incubator settings were maintained at a 5% CO_2_ atmosphere at 37 °C; temperatures were periodically measured using a thermosensitive probe. Cells in chambers were imaged at 2 min intervals for a total of 6 h using a 10× phase objective and the built-in Multi-Dimensional Acquisition function in Micro-Manager. At the onset of imaging, macrophages were stimulated with a combination of 5 ng/ml of *E. coli*-derived UltraPure LPS (Invivogen) with 1 ng/ml of recombinant murine IFN-γ (R&D Systems) or 10 ng/ml of IL-4 (BioLegend) and 10 ng/ml IL-13 (BioLegend). The centers of cell nuclei were annotated using ImageJ's built-in MTrackJ plug-in (imagescience.org).^45^ Cells that divided or migrated out of the imaging frame were considered only up to the time point of division or exit. At least 65 cells were assessed for each condition per biological replicate. Metrics such as velocity and maximum displacement were quantified using a custom Python script. Measurements were further analyzed in R. Statistical analysis was performed using one-way ANOVA followed by Tukey's HSD post-hoc test.

### Enzyme-linked immunosorbent assay (ELISA)

BMDMs were seeded at 100 000 cells/cm^2^ on TCPS, non-crosslinked, crosslinker control, or crosslinked fibrin gels. Macrophages were stimulated at 6 h after seeding with a combination of 5.0 ng/ml of *E. coli*-derived UltraPure LPS (Invivogen) with 1.0 ng/ml of recombinant murine IFN-γ (R&D Systems). At 12 h after stimulation, tumor necrosis factor alpha (TNF-α) and monocyte chemoattractant protein-1 (MCP-1) secretion levels were assessed by enzyme-linked immunosorbent assay (ELISA) following manufacturer's protocol (BioLegend). Statistical analysis was performed using Student's T-test followed by false discovery rate corrections.

### Western blotting

After stimulating BMDMs with LPS and IFN-γ for 18 h, total protein was extracted using radioimmunoprecipitation assay (RIPA) lysis buffer (VWR) supplemented with 1× of Halt protease and phosphatase inhibitor cocktail (Thermo Scientific). 20 *μ*g of total protein was resolved on 4%–15% Mini-PROTEAN TGX precast gels (Bio-Rad). Protein was blotted onto the nitrocellulose membrane using iBlot2 transfer systems (Invitrogen) and then probed using NF-κB and phosphoNF-κB (p65, Cell Signaling Technologies) and GAPDH (Biolegend) antibodies. Statistical analysis was performed considering p < 0.05 to be statistically significant. Data were analyzed using paired Student's t-test assuming equal variance within each surface condition.

### Flow cytometry

Cells were blocked with anti-CD16/32 antibody (clone 2.4G2, Tonbo Biosciences) and then stained using antibodies against αM (M1/70 PE), αV (RMV-7 PE), β2 (M18/2 FITC), and β3 (2C9.G2 APC) integrins from Biolegend. PE-conjugated antibodies against α4, α5, and β1 integrins were obtained from Santa Cruz Biotechnology. Isotype controls were purchased from the corresponding vendor, and Fc block antibody was from Tonbo. For blocking, LEAF-grade antibodies against β1 (HMβ1-1), β2 (M18/2), and β3 (2C9.G2) integrins and matching isotype controls were purchased from Biolegend. Thorough washing was performed to remove excess, unbound antibodies. Flow cytometry was performed on a BD LSRII flow cytometer using BD FACSDiva software (BD Biosciences). Post-processing was performed in FlowJo (Treestar), and further data analysis and quantification was performed in R. Cell populations were gated on forward and side scatter to select for intact, single cells. Acquisition was performed until at least 10 000 events were collected using a preliminary analysis gate or until the sample was exhausted. Statistical analysis was performed considering p < 0.05 to be statistically significant. Data were analyzed using a one-way ANOVA followed by Tukey's HSD post-hoc test.

### Integrin knockdown

Knockdown of integrin genes was performed by nucleofection (4D-Nucleofector system, Lonza) using siRNAs (siGENOME siRNAs, Dharmacon). Briefly, 0.5 × 10^6^ freshly isolated BMDMs were transfected with 100 nM of siRNA in 20 *μ*l of nucleofection solution. After nucleofection, cells were recovered in RPMI-1640 complete media (10% Heat inactivated FBS supplemented with 1% P/S and 10% MCSF) for 24 h and stimulated with LPS and IFN-γ as described above. The supernatant was collected 24 h post stimulation and analyzed for cytokine secretion by enzyme-linked immunosorbent assay (ELISA) following manufacturer's protocol (Biolegend), or live imaging was performed.

### Ethics approval

All procedures involving animals were approved by the University of California, Irvine's Institutional Animal Care and Use Committee (protocol #AUP-17-85), which is accredited by the Association for the Assessment and Accreditation of Laboratory Animal Care International (AAALACi). There were no procedures involving human participants.

## SUPPLEMENTARY MATERIAL

See supplementary material for experiments examining the effects of collagen, further analysis of the aspect ratio and velocity on polarized macrophages, and movies of migrating cells on different substrates.
